# The role of the Hsp90/Akt pathway in myocardial calpain-induced caspase-3 activation and apoptosis during sepsis

**DOI:** 10.1186/1471-2261-13-8

**Published:** 2013-02-20

**Authors:** Xiaoping Li, Rong Luo, Rongjian Jiang, Xianmin Meng, Xiushan Wu, Shu Zhang, Wei Hua

**Affiliations:** 1 Cardiac Arrhythmia Center, State Key Laboratory of Cardiovascular Disease, Fuwai Hospital, National Center for Cardiovascular Diseases, Chinese Academy of Medical Sciences and Peking Union Medical College, Beijing 100037, P.R. China; 2 Department of Cardiology, Sichuan Provincial People’s Hospital, Chengdu 610072, P.R. China; 3 The Center of Heart Development, Key Lab of MOE for Development Biology and Protein Chemistry, College of Life Science, Hunan Normal University, Changsha, Hunan 410081, PR China; 4 Central Laboratory, State Key Laboratory of Cardiovascular Disease, Fuwai Hospital, National Center for Cardiovascular Diseases, Chinese Academy of Medical Sciences and Peking Union Medical College, Beijing 100037, People’s Republic of China

**Keywords:** Calpain, Hsp90/Akt, Caspase-3 activation, Apoptosis, Sepsis

## Abstract

**Background:**

Recent studies have demonstrated that myocardial calpain triggers caspase-3 activation and myocardial apoptosis in models of sepsis, whereas the inhibition of calpain activity down-regulates myocardial caspase-3 activation and apoptosis. However, the mechanism underlying this pathological process is unclear. Therefore, in this study, our aim was to explore whether the Hsp90/Akt signaling pathway plays a role in the induction of myocardial calpain activity, caspase-3 activation and apoptosis in the septic mice.

**Methods:**

Adult male C57 mice were injected with lipopolysaccharide (LPS, 4 mg/kg, i.p.) to induce sepsis. Next, myocardial caspase-3 activity and the levels of Hsp90/p-Akt (phospho-Akt) proteins were detected, and apoptotic cells were assessed by performing the TUNEL assay.

**Results:**

In the septic mice, there was an increase in myocardial calpain and caspase-3 activity in addition to an increase in the number of apoptotic cells; however, there was a time-dependent decrease in myocardial Hsp90/p-Akt protein levels. The administration of calpain inhibitors (calpain inhibitor-Ш or PD150606) prevented the LPS-induced degradation of myocardial Hsp90/p-Akt protein and its expression in cardiomyocytes in addition to inhibiting myocardial caspase-3 activation and apoptosis. The inhibition of Hsp90 by pretreatment with 17-AAG induced p-Akt degradation, and the inhibition of Akt activity by pretreatment with wortmannin resulted in caspase-3 activation in wildtype C57 murine heart tissues.

**Conclusions:**

Myocardial calpain induces myocardial caspase-3 activation and apoptosis in septic mice via the activation of the Hsp90/Akt pathway.

## Background

Endotoxins or bacterial lipopolysaccharides depress myocardial contractility in laboratory animals and humans [1,2]. Although, the molecular and cellular mechanisms that mediate the pathogenesis of septic cardiomyopathy are still unclear, several lines of evidence suggest that myocardial caspase-3 activation plays a major role in myocardial dysfunction [3-6].

The blockade of myocardial caspase-3 activation significantly attenuates myocardial dysfunction and improves the survival rate during sepsis [4-6], and therefore, the mechanism involved in LPS-induced caspase-3 activation has been explored in cardiomyocytes [7-9]. In a recent study that we published, an increase in myocardial calpain activity in the septic mouse was noted [7,8], and in addition, over-expression of calpastatin, a specific inhibitor of calpain, or treatment with pharmacological inhibitors of calpain prevented myocardial caspase-3 activation during endotoxemia. These results suggest that calpain is involved in the activation of caspase-3 during sepsis [7]. However, the mechanisms involved in calpain-induced caspase-3 activation have not been completely defined in septic cardiomyocytes.

Akt, a serine/threonine and prosurvival kinase, is involved in the regulation of caspase-3 activation and apoptosis [10-13]. Heat shock protein 90 (Hsp90), a molecular chaperone, is essential for the proper functioning of Akt because it forms a chaperone-substrate protein complex, and a reduction in Hsp90-Akt binding results in Akt inactivation [14]. Therefore, it is possible that activated calpain induces caspase-3 activation and apoptosis via cleavage of its substrate Hsp90, a key Akt regulator protein, and inhibition of Akt activation [15,16]. Therefore, we hypothesized that calpain activation would adversely affect the Hsp90/Akt signaling pathway and induce caspase-3 activation and apoptosis during sepsis.

In this study, we have determined the role of the Hsp90/Akt pathway in lipopolysaccharide (LPS)-induced myocardial caspase-3 activation and apoptosis. We observed that the inhibition of calpain reduced Hsp90 degradation and increased Akt activity, thereby preventing caspase-3 activation and apoptosis in septic mice. These results indicate that the Hsp90/Akt pathway negatively regulates LPS-induced myocardial caspase-3 activation and apoptosis.

## Methods

### Animal preparation

Pathogen-free and wild-type adult C57BL/6 mice (male, 6–8 weeks, 25–30 g) were used. Animals were housed under a 12 h light–dark cycle with food and water available *ad libitum*. All of the experimental procedures were approved by the Institutional Animal Ethics Committee of Peking Union Medical College.

In this study, a total of 90 mice were divided into six different groups with 15 mice in each group). The control mice (sham group) were injected intraperitoneally (i.p) with 100 μl PBS solution, and the LPS-treated mice were injected with LPS (4 mg/kg, i.p), which was isolated from Escherichia coli serotype 055:B5 (Sigma, St. Louis, MO) and dissolved in 100 μl PBS solution. Calpain inhibitor-Ш (10 mg/kg, i.p) or PD150606 (3 mg/kg, i.p) plus LPS treated mice were injected i.p, and the calpain inhibitors-III or PD150606 were dissolved in 80 μl DMSO. The mice were injected i.p with either calpain inhibitor-III or PD150606 alone 30 minutes before injecting LPS, and all of the mice were subjected to biological and physiological experiments at 4 h post-treatments. In addition, the time course experiments were performed at 0, 1, 2, 4, and 6 h after LPS injection, and 5 mice were used for each time point.

### Calpain activity assay

Calpain activity was measured using the fluorescence substrate, N-succinyl- LLVY-AMC (Cedarlane Laboratories, Burlington, NC, USA), as previously described [17]. This assay measures the fluorescence intensity of AMC when it is cleaved from a peptide substrate. The fluorescence intensity of the cleaved AMC was quantified by using a multilabel reader (excitation, 360 nm; emission, 460 nm, Wallac 1420, PerkinElmer, Turku, Finland), and calpain activity was determined by measuring the difference between calcium-dependent and calcium-independent fluorescence. All experiments were conducted in duplicate.

### Caspase-3 activity assay

Myocardial caspase-3 activity was measured using a caspase-3 fluorescent assay kit according to the manufacturer’s protocol (BIOMOL Research Laboratories) [17]. Briefly, the whole hearts were isolated from mice and homogenized. Duplicate sets of protein samples were incubated with either Ac-DEVD-AMC, a caspase-3 substrate, or Ac-DEVD-AMC plus the inhibitor, ACDEVD-CHO, at 37°C for 2 h before the measurements were obtained by using a fluorescent spectrophotometer (excitation at 380 nm, emission at 405 nm, Wallac 1420, PerkinElmer, Turku, Finland). The signals obtained from the inhibitor-treated samples served as the background.

### Western blotting analysis

The proteins (40 μg each lane) from each sample were subjected to SDS-PAGE using a 10% gel and subsequently electrotransferred onto membranes. The expression levels of Hsp90, p-Akt/Akt and glyceraldehyde-3-phosphate dehydrogenase (GAPDH) proteins were determined by first probing the blots with specific antibodies (1:1000, Cell Signaling, Danvers, MA) and then by performing enhanced chemiluminescence detection.

### *In situ* detection of apoptotic cells

To identify and quantitatively assess the number of cells that underwent apoptosis in the heart, the terminal deoxynucleotidyl transferase (TdT)-mediated dUTP nick-end labeling (TUNEL) assay was performed on the paraffin-embedded sections of murine heart tissues using an *in situ* apoptosis detection kit (Roche Molecular Biochemicals), according to the manufacturer’s instructions, based on our previous report [18]. All of the sections were analyzed using a Leica microscope.

### Histological preparation and immunohistochemistry

The murine heart tissues were fixed in a 4% paraformaldehyde-PBS solution over a period of 24 h and subjected to standard histological procedures for paraffin-embedded sections. Paraffin sections (5 μm thickness) were sliced for performing immunohistochemical experiments and incubated with rabbit anti-mouse Hsp90 and p-Akt antibodies (1:100, Cell Signaling) overnight at 4°C. The expression levels of Hsp90 and p-Akt in the heart tissues were visualized by employing routine immunoperoxidase techniques. The sections were first counterstained with hematoxylin and then dehydrated and mounted using routine methodologies. The secondary goat polyclonal antibody (IgG) was purchased from Jackson (USA).

### Statistical analysis

All of the data are presented as the mean ± SE. The statistical differences between the two groups were compared using unpaired Student’s *t*-test. One-way ANOVA followed by the Student-Newman-Keuls test was used for performing multigroup comparisons. *P* < 0.05 was considered to be statistically significant.

## Results

### Myocardial calpain activities increased in septic mice

**Figure 1:**
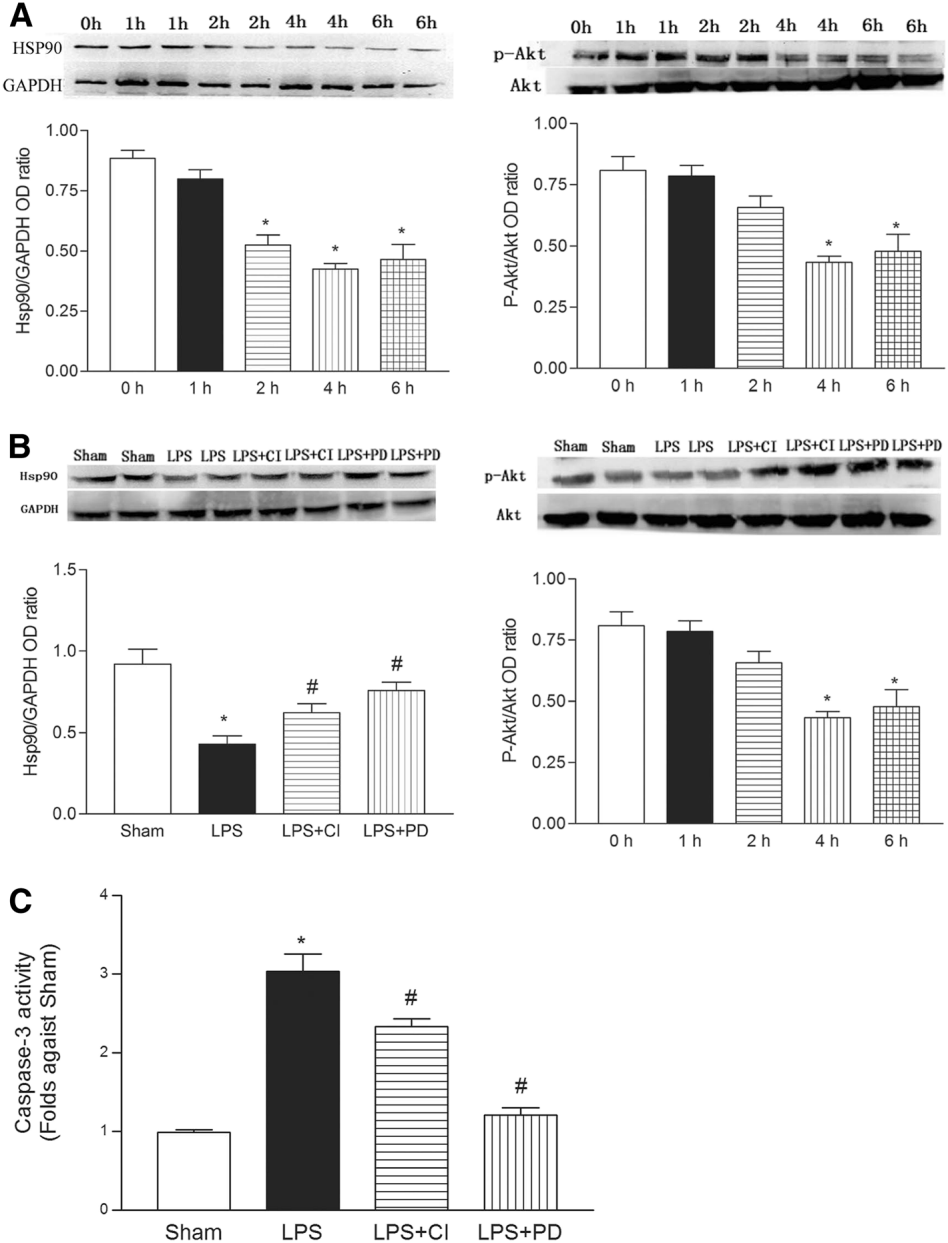
**Panel A, The time course of myocardial Hsp90/p-Akt (Thy308) protein expression.** Hsp90 and p-Akt/Akt protein expression was determined by Western blot at 0, 1, 2, 4, and 6 h after LPS (4 mg/kg) treatment. Panel **B**, Myocardial Hsp90/p-Akt expression is decreased in septic mice. In the mice were treated with LPS (4 mg/kg, i.p.) for 4 h, the myocardial Hsp90/p-Akt expression was detected. Panel **C**, Myocardial caspase-3 activity in the septic mice. Mice were treated with LPS (4 mg/kg, i.p.) for 4 h, the heart tissues were extracted and caspase-3 activity was determined. The data are shown as the mean ± SE (n = 5). LPS, lipopolysaccharide; CI, calpain inhibitor-III., PD, PD150606. * vs. sham, *P* < 0.05; # vs. LPS, *P* < 0.05.

In the present study, mice were first injected with either calpain inhibitor-III (10 mg/kg, i.p) or PD150606 (3 mg/kg, i.p), and 30 minutes later, LPS was injected (4 mg/kg, i.p) to establish a model of sepsis. As reported in our previous study, 4 h after LPS injection, the increase in myocardial calpain and caspase 3 activity in the septic mice were compared with that observed in the control mice [7]. Both calpain inhibitor-III and PD150606 significantly inhibited the increase in myocardial calpain activity (data not shown) and caspase-3 activation (Figure 1C) in septic mice. Neither calpain inhibitor-III nor PD150606 alone had an obvious effect on the myocardial calpain activity in wild type mice (data not shown).

### Decrease in the myocardial p-Akt and Hsp90 proteins in LPS-challenged C57 mice

The Hsp90/Akt pathway is a well-known signaling pathway that is anti-apoptotic and promotes cell survival in a variety of conditions including sepsis [16,19-22]. It has been recently demonstrated that calpain decreased phospho-Akt (p-Akt, Thy308) levels and inhibited the Akt pathway [16]. In the LPS-challenged C57 mice, the total Akt protein content did not change; however, there was a significant time-dependent reduction in the amounts of p-Akt and Hsp90, the decrease in myocardial Hsp90/p-Akt expression was maximal at approximately 4 h. Blockade of calpain activation by either calpain inhibitor-β or PD150606 prevented the down-regulation of Hsp90 protein and promoted Akt activation; this was demonstrated by an increase in p-Akt protein levels (Figure 1A, B). Therefore, these results indicate that the observed decrease in Hsp90/p-Akt protein was mediated by calpain. Meanwhile, the caspase-3 activity was accordingly increased in the LPS-challenged mice (Figure 1C), and the calpain inhibitors, calpain inhibitor-Ш and PD150606, prevented the activation of caspase-3.

### Localization of Hsp90/p-Akt protein in the myocardium

**Figure 2:**
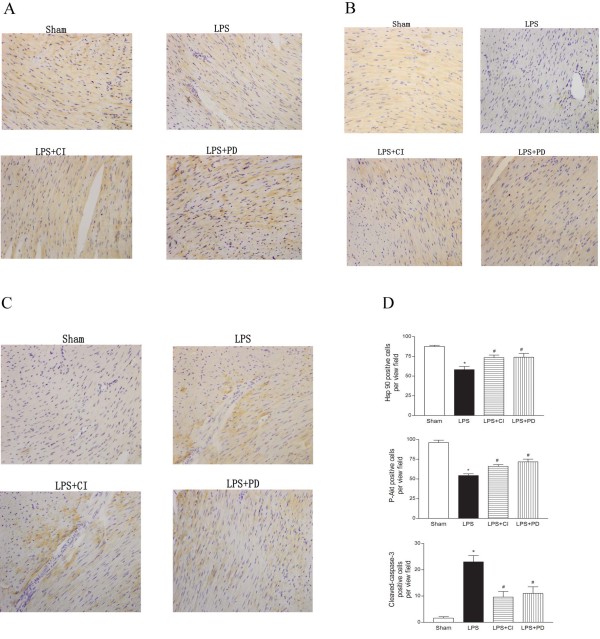
**The representative immunohistochemical photomicrographs of Hsp90/p-Akt (Thy308).** The positive expression of Hsp90 was indicated by brown staining (original magnification × 400). Representative immunohisto- chemical data depicting cardiac Hsp90/p-Akt and cleaved caspase-3 expression in the LPS-treated mice (×400). Panels **A** and **B**, Representative pictures reveal a significant decrease in Hsp90 (A) and p-Akt (B) positive expression in the cytoplasm of the myocytes after LPS treatment. Panel **C**, Representative pictures of cleaved caspase-3 (the antibody was specific to detect the large fragment 17/19 KDa of activated caspase-3) expression in the cytoplasm after LPS treatment or LPS plus calpain inhibitors, calpain inhibitor III and PD150606 in the septic mice. Panel **D**: Representative quantitative data of Hsp90/p-Akt and cleaved caspase-3 expression in the septic mice and after treatment with calpain inhibitors. The values were from ten high power fields each section randomly selected and counted manually. CI, calpain inhibitor-III; PD, PD150606. * *P* < 0.05 vs. sham,# *P* < 0.05 vs. LPS.

Immunohistological staining of the myocardial tissues was performed to evaluate the expression of Hsp90 and Akt in the myocardium. As demonstrated in Figure 2, after LPS stimulation for 4 h, there was a significant decrease in the number of cardiomyocytes that were positive for Hsp90 and p-Akt staining in the cellular cytoplasm; however, co-treatment with either calpain inhibitor-Ш or PD150606 increased Hsp90 and p-Akt staining in the myocardium (Figures 2A and B). These results further confirmed that cardiomyocytes were the major source of Hsp90 and Akt in the myocardium, and that blocking calpain activation restored the expression of Hsp90 and Akt in response to LPS treatment.

### Myocardial caspase-3 activation and apoptosis in the septic heart

**Figure 3:**
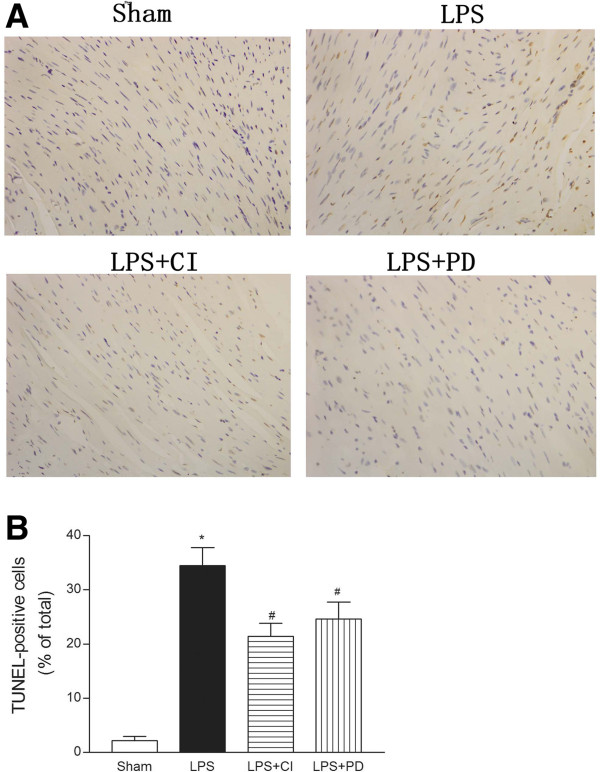
**Determination of apoptosis in the cardiomyocytes.** Panel **A**, Four hours after LPS treatment (4 mg/kg), 4-mm paraffin- embeded tissue sections were prepared from LV apex and TUNEL staining of the nuclei of cardiomyocytes from control and LPS-treated mice was performed. Panel **B**, The apoptosis index among the sham-treated mice and mice treated with LPS or LPS plus calpain inhibitors is demonstrated. CI, calpain inhibitor-III; PD, PD150606. * *P* < 0.05 vs. sham, # *P* < 0.05 vs. LPS (n = 5).

Immunohistological staining of the myocardial tissues revealed that a cleaved form of caspase-3 was expressed in the septic mice. Further, the inhibition of calpain by the calpain inhibitors reduced the expression of the cleaved caspase-3 (Figure 2C). In addition, apoptotic cardiomyocytes were detected by TUNEL staining in the myocardia of septic mice. The ratio of TUNEL-positive cardiomyocytes to the total number of cardiomyocytes in the samples obtained from LPS plus calpain inhibitor-III or PD150606 group was significantly reduced compared to the LPS group (36.4 ± 8.15% versus 22.5 ± 4.42%, 26.2 ± 6.75%, respectively, n = 5, p < 0.01) (Figure 3). These findings indicate that calpain inhibitors protect the cardiomyocyte against LPS-induced apoptosis.

### Blockade of Akt by the PI3K inhibitor induces caspase-3 activation

**Figure 4:**
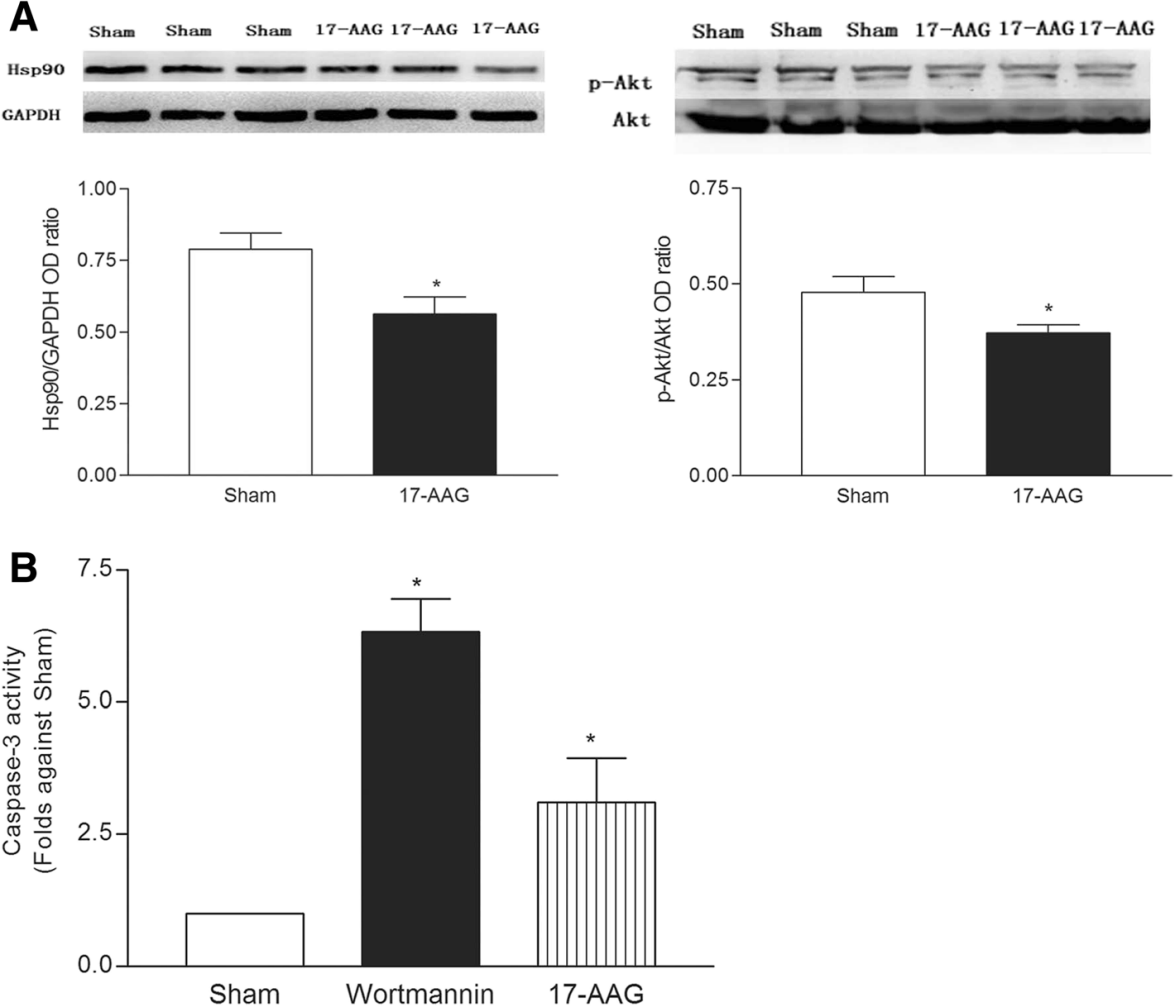
**The effects of treatment with 17AAG (Hsp90 inhibitor) on Akt activity and wortmannin (PI3K inhibitor) on caspase-3 activation are depicted.** Mice were treated with either 17-AAG or wortmannin (3 mg/kg, i.p). Four hours after administration, the activation of Akt and caspase-3 was detected. * *P* < 0.05 vs. sham (n = 5).

To detect the role of Hsp90 in the activation of myocardial caspase-3, we used the Hsp90-selective inhibitor, 17-allylamino-17-demethoxygeldanamycin (17-AAG), to inhibit the Hsp90 activity in C57 wildtype mice. Blockade of Hsp90 activity by 17-AAG (30 mg/kg, i.p) produced a decrease in p-Akt protein and caspase-3 activation (Figures 4A and B). In addition, treating the C57 mice with wortmannin (3 mg/kg, i.p), an inhibitor of PI3 kinase (the enzyme responsible for Akt phosphorylation), prevents the activation of Akt by inhibiting Akt phosphorylation with PI3 kinase, which is observed with the increase in caspase-3 activity (Figures 4A and B). These data suggest that the Hsp90/Akt-dependent signaling pathway down-regulates the apoptotic signaling pathway in myocardial tissues.

## Discussion

To our knowledge, this is the first study that suggests the existence of a direct link between the activation of calpain and the subsequent activation of caspase-3 with the Hsp90/Akt pathway. We observed that myocardial calpain induces caspase-3 activation and apoptosis. The underlying mechanism involves a myocardial calpain-induced decrease in Hsp90/p-Akt protein levels and inhibition of Akt signaling, which increases caspase-3 activity and apoptosis during sepsis.

It has been demonstrated that calpain activation during sepsis [7-9,17,23] plays an important role in caspase-3 activation and cellular apoptosis via the cleavage of pro- or anti-apoptotic proteins [24,25]. It was observed that caspase-3 activation was involved in the process of myocardial dysfunction via the cleavage of α-actin, α-actinin, and TnT, which produced direct functional effects on the myofilament activation and contractile function [26]. However, in our limited study we failed to notice the cleavage of Bcl-2 and Bad in response to myocardial calpain activation in the septic mouse [8].

Akt, a serine/threonine kinase, is downstream of phosphatidylinositol 3-kinase (PI3 kinase) and is involved in the regulation of caspase-3 activation and apoptosis. This enzyme becomes phosphorylated and activated by a number of growth factors, cytokines and hormones, inhibits caspases and exerts anti-apoptotic effects by inactivating GSK-3β [27], the latter activating p53 [28], inducing stress to the endoplasmic reticulum [29], phosphorylation and translocation of Bax to the mitochondria [30]. In addition, Akt inhibits the activation of caspases and apoptosis by inhibiting Bid (the deactivator of caspase-9) and retaining cytochrome c in the mitochondria [10,12,13]. In our laboratory, we observed that there was a decrease in the level of p-Akt protein in LPS-treated cardiomyocytes [19]. In this study, we observed that in the septic mice, calpain was activated and p-Akt was decreased. Further, the inhibition Akt signaling by wortmannin induced myocardial caspase-3 activation in wildtype C57 mice. These data indicate that Akt signaling plays an important role in the activation of myocardial caspase-3 during sepsis.

To investigate potential mechanisms of calpain-mediated Akt inhibition, we next determined whether calpain activation altered Hsp90 protein content and/or the interaction between Hsp90 and Akt proteins. Akt is one of Hsp90’s substrates, and therefore, Hsp90 contributes to the functional stabilization of Akt, activation of PI3K/Akt signaling pathway and cell survival. In addition, Hsp90 regulates Akt activity by inhibiting its dephosphorylation and proteosomal degradation [31,32]. The Hsp90/Akt pathway is an important survival and antiapoptic pathway in a variety cells and settings because the cleavage of Hsp90 in Akt/Hsp90 complex appears to be very important in the destabilization of the Akt/Hsp90 complex and in the triggering of apoptotic signals [20-22]. As Hsp90 has been demonstrated to be a substrate of calpain in the diaphragm muscle of the rat, calpain activation by supplementation with Ca^2+^ in vitro led to the cleavage of Hsp90 and caused inhibition of the Akt signaling pathway [16]. These results suggest that calpain activation may diminish Hsp90-Akt binding and consequently inactivate the Akt signaling pathway. In this study, the expression levels of the myocardial Hsp90 protein were decreased in response to calpain activation, suggesting that myocardial calpain cleaved Hsp90, which then induced p-Akt degradation and inhibition of Akt signaling in septic mice.

## Conclusion

In this study, we found that the Hsp90/Akt signaling pathway plays a role in the induction of myocardial calpain activity, caspase-3 activation and apoptosis in the septic mice. The activated calpain induces caspase-3 activation and apoptosis via cleavage of Hsp90, an Akt molecular chaperone protein, and inhibition of Akt activation indicated by the decrease in myocardial p-Akt protein levels, which induces caspase-3 activity and apoptosis during sepsis.

### Key messages

•Myocardial calpain and caspase-3 activity increased in the septic mice.

•There was a time-dependent decrease in myocardial Hsp90/p-Akt protein levels in the septic mice.

•Calpain inhibitors prevented the LPS-induced degradation of myocardial Hsp90/p-Akt protein and its expression in cardiomyocytes

•The inhibition of Hsp90 by pretreatment with 17-AAG induced p-Akt degradation.

•The inhibition of Akt activity by pretreatment with wortmannin resulted in caspase-3 activation in wildtype C57 murine heart tissues.

## Abbreviations

LPS: Lipopolysaccharide;Hsp90: Heat shock protein 90;GAPDH: Glyceraldehyde-3-Phosphate Dehydrogenase;TUNEL: Tdt-Mediated Dutp Nick-End Labeling

## Competing interests

The authors declare that they have no competing interests.

## Authors’ contributions

LX and LR carried out the Animal preparation, immunohistochemistry and calpain activity assay; they also contributed to analysis and interpretation of data. JR and MX carried out caspase-3 activity assay and Western blotting analysis. WX and ZS participated *in situ* detection of apoptotic cells and drafted the manuscript. LX and HW designed, set up and monitored the study. All authors read and approved the final manuscript.

## Pre-publication history

The pre-publication history for this paper can be accessed here:

http://www.biomedcentral.com/1471-2261/13/8/prepub
